# Health Care–Associated Infection Outbreak Investigations in Outpatient Settings, Los Angeles County, California, USA, 2000−2012

**DOI:** 10.3201/eid2108.141251

**Published:** 2015-08

**Authors:** Kelsey OYong, Laura Coelho, Elizabeth Bancroft, Dawn Terashita

**Affiliations:** Los Angeles County Department of Public Health, Los Angeles, California, USA (K. OYong, D. Terashita);; Centers for Disease Control and Prevention, Atlanta, Georgia, USA (L. Coelho);; Independent Consultant, Los Angeles (E. Bancroft)

**Keywords:** ambulatory care facilities, disease outbreaks, outbreak investigations, communicable disease control, infection control, outpatient settings, health care, viruses, bacteria, fungi, parasites, Los Angeles County, California, United States

## Abstract

Health care services are increasingly delivered in outpatient settings. However, infection control oversight in outpatient settings to ensure patient safety has not improved and literature quantifying reported health care–associated infection outbreaks in outpatient settings is scarce. The objective of this analysis was to characterize investigations of suspected and confirmed outbreaks in outpatient settings in Los Angeles County, California, USA, reported during 2000–2012, by using internal logs; publications; records; and correspondence of outbreak investigations by characteristics of the setting, number, and type of infection control breaches found during investigations, outcomes of cases, and public health responses. Twenty-eight investigations met the inclusion criteria. Investigations occurred frequently, in diverse settings, and required substantial public health resources. Most outpatient settings investigated had >1 infection control breach. Lapses in infection control were suspected to be the outbreak source for 16 of the reviewed investigations.

## Abstract 2

Most investigations identified a control breach as the source of infections.

Health care services are increasingly delivered in outpatient settings rather than inpatient, acute-care settings. Nationwide, nearly 1.2 billion outpatient visits occur per year (*1*). Outpatient facilities encompass a broad array of facilities, such as primary care clinics, ambulatory surgery centers, pain clinics, oncology clinics, imaging facilities, dialysis centers, urgent care centers, and other specialized facilities. The types of procedures performed in outpatient settings are also diverse and include myriad procedures, from podiatry and nail clipping to advanced surgeries (e.g., joint replacements).

Procedures performed in outpatient settings are often invasive and carry risks of infection. Many of these procedures were previously performed in hospitals in which infection control practices are subject to regular oversight and regulation (*2*). Despite the increase in ambulatory care, there has not been a corresponding increase in infection control oversight in outpatient settings, and data are insufficient on the rates of infections resulting from procedures performed in outpatient settings (*3*).

At the same time, the amount of literature reporting a need for infection control oversight in outpatient settings is increasing. For example, during 2001–2011, there were >18 outbreaks of viral hepatitis associated with unsafe injection practices in outpatient settings, such as physician offices or ambulatory surgery centers (*4*). In addition, in an infection control audit conducted by the Centers for Medicare and Medicaid Services (CMS) in 2008, a total of 46 (68%) of 68 ambulatory surgery centers surveyed had >1 lapse in infection control; 12 (18%) had lapses identified in >3 of 5 infection control categories (*5*). CMS now requires adherence to its infection control surveyor worksheet for participation in CMS by ambulatory surgery centers (*6*). However, many outpatient settings opt out of participation in CMS or are not licensed by state health departments and are thus not held to the standardized infection control standards.

We recognized the infection control concerns associated with outpatient settings. Therefore, the Los Angeles County Department of Public Health (LACDPH) conducted an analysis to characterize health care–associated infection (HAI) outbreak investigations in Los Angeles County outpatient settings.

## Databases and Study Population

An outpatient setting is defined as a distinct health care entity, either hospital-based or nonhospital-based, that operates exclusively on an outpatient basis for patients who do not require hospitalization and who are expected to stay <24 hours.

In California, all outbreaks, confirmed or suspected, are mandated to be reported to the respective local health department. We defined an outbreak as a suspected occurrence of cases of a disease above the expected or baseline level, over a given period of time, in a geographic area or facility, or in a specific population group (*7*). This review analyzed all HAI outbreak investigations that occurred in an outpatient setting, which included some investigations that found no cases but instead were enacted as an inquiry into deficient medical practices. The LACDPH is responsible for investigating communicable disease outbreaks in this county, as reported by general acute care hospitals, outpatient settings, schools, residential facilities, and other sources. Reported outbreaks are documented in the LACDPH disease control outbreak log (outbreak log) with details of the investigation, suspected source, and number of cases.

To identify suspected and confirmed HAI outbreaks that occurred in outpatient settings and which were investigated by the LACDPH during January 2000–December 2012, we reviewed the outbreak log database and other internal publications, records, and correspondence. Outbreaks were classified as confirmed if the implicated infectious disease agent was verified by laboratory findings. Outbreaks were classified as suspected when such findings could not be verified. We classified outbreak investigations by oversight (licensure and accreditation status), hospital affiliation, and type of setting. Settings that were licensed by the California Department of Public Health Licensing and Certification Division at the time of the outbreak investigation were considered to be licensed. Settings with documented accreditation at the time of the investigation by an accrediting organization recognized by the California Medical Board were considered to be accredited. Outpatient settings affiliated with a hospital were under the common ownership, licensure, or control of the hospital (*8*). Ophthalmology offices, hospital clinics, urology offices, radiology offices, pain clinics, orthopedist offices, oncology offices, obstetrics/gynecology clinics, and medical spas were grouped together into offices/clinics.

Suspected and confirmed HAI outbreak investigations were classified by number and type of infection control breaches, time until reporting, duration of investigation, and number and outcome of cases. The time until reporting of an outbreak was defined as time from onset of the first case or the time from first known exposure to the breach until notification of the Los Angeles County Department of Public Health. Duration of investigation was defined as time from notification of the LACDPH to closure in the outbreak log.

Public health response was separated into several categories, including site visit(s), medical record review, patient notification, active surveillance, recommendations to facility, sample collection, laboratory analysis, and environmental investigation. Patient notification refers to the process of informing patients about potential exposures through mailed notification letters or postage of a letter in the facility. Active surveillance is surveillance in which the LACDPH proactively solicited infection reporting (e.g., analyzed current patient medical records from facilities for case finding or surveying patients to identify additional cases).

Sample collection involved the ascertainment of biologic specimens from patients (e.g., from blood, wound, urine), environmental samples (e.g., water, air), medication samples, and samples from equipment (swab specimens from inside or outside equipment). Laboratory analyses included genetic typing, pulsed-field gel electrophoresis for DNA fingerprinting, and genomic sequencing. Laboratory analysis was either conducted by the LACDPH Laboratory or sent to the Centers for Disease Control and Prevention (CDC) laboratory or the California Department of Public Health laboratory for testing. Environmental investigations were conducted in conjunction with the LACDPH Environmental Health Division and involved evaluating facility layouts and environmental infection control procedures.

Infection control characteristics were classified into 10 categories. These categories included breaches in hand hygiene, use of personal protective equipment, injection safety, medication documentation, equipment processing and sterilization, written infection control policies and procedures, and staff credentials. Because all investigations were undertaken conducted as outbreak investigations under public health authority and considered routine public health activities, this analysis was exempt from institutional review board review.

## Measures

We examined outbreak investigations by setting type, oversight, infection control findings, duration, and outcome of cases. The criterion for statistical significance was a 2-sided χ^2^ test p value <0.05. Analyses were conducted by using SAS statistical software version 9.3 (SAS Institute Inc., Cary, NC, USA).

## Characterization of Outbreak Investigations

Twenty-eight investigations of HAI outbreaks in outpatient settings in Los Angeles County met the inclusion criteria; 22 were classified as confirmed outbreaks and 6 were classified as suspected outbreaks. The summary characteristics for the 28 investigations are shown in Table 1.

**Table 1:** Attributes of 28 selected health care–associated infection outbreaks in outpatient settings, Los Angeles County, California, USA, 2000–2012*

Setting type	Year investigation started	No. cases	Suspected agent type	Suspected agent	Comment
Dialysis center	2001	6	Bacterial	*Enterobacter cloacae*	Hemodialysis machines not maintained and inner tubing visibly contaminated (*9*)
Oncology office	2001	11	Bacterial	*Alcaligenes xylosoxidans*	Outbreak associated with reuse of contaminated vial of heparin or saline
Dialysis center	2002	7	Bacterial	MRSA	Hypothesized contamination of medicine by preparation in patient care area
Ophthalmologist office	2002	15	Bacterial, viral	*Streptococcus pneumoniae,* adenovirus	Investigation of conjunctivitis; possible transmission by health care workers’ hands, breaks in aseptic technique, and use of multidose vials
Dialysis center	2002	36	Bacterial	Mixed infection	Increased infection rate in 2 dialysis centers prompted study; associated infections with improperly disinfected reused dialyzers
Dialysis center	2003	4	Bacterial	*Stenotrophomonas maltophilia*	Inadequate sterilization of dialyzers and endotoxin in reverse osmosis water
Residential facility/ retirement center	2004	4	Viral	HBV	Hand hygiene deficiencies noted for nurses performing fingersticks to monitor blood glucose (*10*)
Dialysis center	2004	14	Bacterial	*Serratia marcescens*	Cluster of infections among mostly dialysis patients; no common source identified
Plastic surgeon	2004	0	Other	NI	Cosmetic surgeon was collecting and transplanting cartilage without proper consent, storage or donor testing; no infections identified with practice
Eye/ophthalmologist office	2007	4	Toxin	NI	Four cases of endophthalmitis in postcataract outpatient surgery patients
Urology office	2007	3	Bacterial	Multiple bacterial organisms	Infections in cystoscopy patients with widespread bacterial contamination of cytoscope and facility
Clinic	2007	3	Ectoparasite	*Sarcoptes scabei*	Facility staff and patients notified by letter of scabies exposure
Skilled nursing facility with contracted home health agency	2008	9	Viral	HBV	Contracted podiatrist used contaminated instruments and infrequently disinfected treatment area (*11*)
Plastic surgeon	2008	1	Bacterial	*Mycobacterium chelonae*	Isolated case; several infection control breaches related to cleaning and disinfection of liposuction equipment and medicine preparation (*12*)
Clinic/OB-GYN	2009	2	Chemical	Lidocaine	Severe reactions to lidocaine received during abortion procedure; suggested error in medicine dosing
Radiology office	2009	5	Bacterial	MRSA	Suggested poor aseptic technique during medication preparation as cause of joint infections
Clinic	2010	2	Bacterial	*Pseudomonas aeruginosa*	Same bronchoscope was used on both patients; possible contamination
Assisted living facility with contracted home health agency	2010	3	Viral	HBV	Three patients with type 1 diabetes serviced by home health agency
Pain clinic	2010	2	Viral	HCV, HBV	Cross-contamination of multidose vial of saline hypothesized as source
Medical spa	2010	1	Bacterial	NI	Cellulitis developed after injections of a cosmetic filler
Assisted living facility with contracted home health agency	2011	2	Viral	HBV	Infection control issues related to fingerstick practices and podiatric care indicated as 2 common risk factors
Assisted living facility with contracted home health agency	2011	1	Viral	HBV	Home health agency contracted 2 nurses to perform fingerstick blood glucose monitoring
Orthopedist office	2011	3	Bacterial	*Staphylococcus aureus*	Orthopedist reported 3 patients who received joint injections with multidose vials
Dialysis center	2011	3	Bacterial	Mixed bacteria	Reprocessing of multiuse dialyzers associated with cases and found to be insufficiently disinfected
Ambulatory surgery center/ ophthalmologist office	2012	15	Fungal	*Fusarium incarnatum-equiseti* species complex and *Bipolaris hawaiiensis*	Cases part of multistate outbreak associated with contaminated compounded products (*1*)
Ambulatory surgery centers	2012	3	Fungal	*Rhizopus* spp.	Cases of postoperative wound infections from different outpatient surgical centers (*13*)
Ambulatory surgery center	2012	0	Fungal	*Aspergillus fumigatus* and *Exserohilum rostratum*	Patients received recalled lots of steroid from compounding pharmacy implicated in a multistate outbreak of fungal meningitis and septic arthritis
Plastic surgeon	2012	7	Bacterial	*Mycobacterium fortuitum*	Use of a can opener to open medication vial and kitchen-grade microwave to warm saline

*MRSA, methicillin-resistant *Staphylococcus aureus*; HBV, hepatitis B virus; NI, not identified; OB-GYN, obstetrics and gynecology; HCV, hepatitis C virus.

Most identified outbreak investigations were in facilities not affiliated with a hospital (20, 71.4%). The most common settings for outbreak investigations were ambulatory surgery centers (6, 21.4%) and dialysis centers (6, 21.4%). Almost half (13, 46.4%) of the outbreak investigations occurred in settings that were licensed by the California Department of Public Health Licensing and Certification Program. We identified only 2 (7.1%) outpatient settings that were accredited at the time of the investigation, but accreditation history was infrequently documented and was difficult to ascertain. The distribution of outbreak investigations by setting type is shown in Table 2.

**Table 2:** Distribution of selected health care–associated infection outbreaks in outpatient settings, by hospital affiliation and setting type, Los Angeles County, California, USA, 2000–2012

Setting	No. outbreak investigations (%)	No. cases (%)
Licensed by state		
Yes	13 (46.4)	111 (66.1)
No	15 (53.6)	57 (33.9)
Hospital affiliation		
Yes	8 (28.6)	42 (25.0)
No	20 (71.4)	126 (75.0)
Type		
Office/clinic	11 (39.3)	53 (31.5)
Ambulatory surgery center	6 (21.4)	26 (15.5)
Dialysis center	6 (21.4)	70 (41.7)
Contracted home health agency	5 (17.9)	19 (11.3)

Outbreaks were reported 0–1,160 days after onset of the first case or exposure of the first case (median 69 days). The longest delay in reporting came from an investigation into a cosmetic surgeon who had been collecting and transplanting cartilage without proper consent, storage, or donor testing. The total case-patient count was 168 (mean 6/outbreak, range 0−36); 59 case-patients (35.1%) were hospitalized, and 5 case-patients (3%) died. Bacterial agents were implicated in 50% (14) of identified outbreak investigations. Viral and fungal agents were the next most common agents implicated in these investigations.

## Public Health Response

Investigations lasted a median of 36 days (range 7–94 days). The most common actions taken by the LACDPH were conducting one or more site visits (78.6% of investigations); providing written recommendations to the facility (78.6%); medical record reviews of cases and other patients (75%); formal interviews of facility staff (64.3%); and laboratory analysis (60.7%). Acute Comunicable Disease Control also often consulted CDC (50.0%) and the California Department of Public Health (35.7%) during investigations. Other outside partners consulted included the Food and Drug Administration, the Medical Board of California, and the California Board of Pharmacy. Patients who did not have cases were notified of possible risk in 7.1% of investigations. In 1 investigation, nearly 2,300 patients were notified of possible exposure. Public health response by the LACDPH is summarized in Table 3.

**Table 3:** Public health response during outbreak investigations, Los Angeles County, California, USA, 2000–2012

Public health response activity	No. (%) outbreak investigations
Site visit	22 (78.6)
Medical record review	21 (75.0)
Formal staff interviews	18 (64.3)
Sample collection	13 (46.4)
Environmental sample*	9 (32.1)
Biologic specimen (patient)	6 (21.4)
Medication sample	4 (14.3)
Laboratory analysis	17 (60.7)
Los Angeles County Public Health Laboratory	14 (50.0)
Centers for Disease Control and Prevention	9 (32.1)
Environmental health investigation	7 (25.0)
Patient interviews	6 (21.4)
Patient notification	2 (7.1)
Active surveillance	8 (28.6)
Review of facility policies and procedures	15 (53.6)
Written recommendations to facility	22 (78.6)

*Includes air, water, and equipment isolates.

## Infection Control Breaches

Of the 28 outbreak investigations, 22 (78.6%) had >1 infection control breach identified (Table 4). The mean number of infection control breaches identified by the LACDPH during the outbreak investigations was 2.4 (range 0–8). The most common breaches recorded were associated with injection safety (10, 35.7%), equipment processing and sterilization (10, 35.7%), medication documentation (7, 25.0%), and environmental cleaning (6, 21.4%). Injection safety violations included reuse of single-dose medications and not using aseptic technique to enter multidose vials. In 2010, the LACDPH investigated a hepatitis C infection that occurred at an unlicensed pain clinic in which the patient had received epidural injections from a multidose saline vial accessed with a needle that had been used on a previous patient who had hepatitis C. Breaches in equipment processing and sterilization included incomplete disinfection of reusable dialyzers after dialysis and use of incorrect cleanser and disinfection method for endoscopes. In an investigation at a urology office, it was discovered that the facility had been improperly cleaning and disinfecting cystoscopes for >10 years.

**Table 4:** Infection control breaches noted in outbreak investigations, Los Angeles County, California, USA, 2000–2012

Infection control breach	No. (%) outbreak investigations
Injection safety	10 (35.7)
Injection preparation technique and environment	7 (25.0)
Single-use medication policies	2 (7.1)
Logging exposure events	2 (7.1)
Equipment processing and sterilization	10 (35.7)
Log of equipment maintenance	2 (7.1)
Documentation or manuals for equipment	2 (7.1)
Medication documentation	7 (25.0)
Dosage or lot number	3 (10.7)
Open date or expiration date	5 (17.9)
Environmental cleaning	6 (21.4)
Hand hygiene	5 (17.9)
Personal protective equipment	3 (10.7)
Proper glove use	2 (7.1)
Documentation of infection control policies and procedures	5 (17.9)
Credentials of staff	5 (17.9)
Single-use equipment (e.g., blood glucose meters)	4 (14.3)
Knowledge and adherence to policies and procedures	4 (14.3)

## Suspected Sources of Outbreaks

Lapses in infection control were suspected as the source for 16 (57.1%) of the outbreak investigations reviewed. Suspected causes included single-use medication used on multiple patients, reuse of finger stick blood glucose meters on multiple patients, deficiencies in dialyzer reprocessing, and improper equipment cleaning and disinfection. Two (7.1%) outbreak investigations identified externally contaminated medication as the suspected source; these medications were manufactured at compounding pharmacies. Nine (32.1%) investigations did not identify a source of the outbreak. One suspected outbreak investigation found no cases and thus identified no source.

## Oversight of Settings and Infection Control

The number of infection control breaches in unlicensed and licensed settings was determined. We found that mean number of infection control breaches was significantly higher when identified in unlicensed versus licensed settings. (3.3 vs. 1.3 breaches; p<0.001).

## Conclusions

The LACDPH documented considerable illness and death associated with the 28 suspected and confirmed HAI outbreak investigations in outpatient settings included in this study. Cumulatively, more than one third of case-patients associated with these investigations were hospitalized; the mortality rate for these case-patients was 3%. Deaths and illnesses attributed to HAIs are largely considered preventable, which makes these high rates a critical concern (*14*).

Analysis showed diversity in types of outpatient and outbreak settings in Los Angeles County. A dozen different types of outbreak settings were identified, including surgery centers with multiple operating rooms; small medical spas and pain clinics; and home-like settings in assisted living centers with visiting nurses. All of these settings performing a variety of services. In addition, we demonstrated that outbreak investigations require substantial public health resources. The 28 investigations required considerable public health response, including site visits, laboratory analysis, and patient notification; the investigations lasted, on average, >1 month.

We found that infection control breaches served as the suspected source of most outbreaks. The average investigation identified several infection control lapses. The most common infection control lapses identified are consistent with those found by a national audit of ambulatory surgery centers (*5*). In a 1991 analysis of outbreaks in outpatient settings and emergency departments during 1961–1990, most identified sources were related to contaminated medical equipment and multidose medication use (*15*). Our analysis found injection safety violations and equipment cleaning issues were the most frequent sources of outbreaks. Contamination of multidose vials and reuse of syringes were common infection control breaches in Los Angeles County during 2000–2012, similar to findings of Goodman and Solomon in 1991 (*15*). The lack of change in 5 decades related to outbreak source and infections resulting from preventable unsafe behaviors is alarming. Most outbreaks documented could have been prevented by using standard precautions and practicing basic infection control (*14**,**16*). These findings highlight a need for more infection control oversight of outpatient settings, as well as better reporting from outpatient settings.

There were limitations to this analysis. This retrospective review relied on availability and completeness of investigation documents. It is possible that some investigations were not documented in the outbreak log or recalled by LACDPH personnel and consequently not included in this review. Accreditation history was difficult to obtain and was incomplete because accreditation status was not collected upon initial investigation and records from the Medical Board of California do not include settings that are now closed. Passive HAI surveillance in outpatient settings in Los Angeles County partly contributed to delayed report to the LACDPH because it heavily relies on facilities to identity and report outbreaks and conditions. The median time between onset of the first case or exposure of the first case and report to the LACDPH was 69 days; some reported years following the first exposure. It is not unusual for detection and investigation of an outbreak to occur several months or even years after the event (*17*). Delayed reporting might be a result of difficulty in tracking infections in outpatient populations (*18*). As a result of reporting issues, the findings of this study might be an underestimation of illness and death associated with HAIs in outpatient settings in Los Angeles County.

Many outpatient settings have limited patient follow-up; therefore, an outbreak in a particular outpatient facility may not be properly recognized and reported to public health authorities. Once an outbreak is identified, reporting to the LACDPH often does not follow protocol partially because some facilities are unaware of the protocol or fear repercussions of an investigation. Delayed reporting can have serious consequences for public health intervention and patient safety because it hampers the ability of public health officials to perform timely investigations and halt an outbreak. To improve reporting of postprocedure infections, outpatient settings should be encouraged to use standardized, validated reporting tools when applicable.

The National Healthcare Safety Network is a useful system for active and passive surveillance of HAIs and can be applied to outpatient settings. Public health officials are encouraged to support the expansion of HAI surveillance and be vigilant in reaching out to and educating outpatient facilities on reporting requirements.

Finally, CDC and the Healthcare Infection Control Practices Advisory Committee created the Guide to Infection Prevention in Outpatient Settings: Minimum Expectations for Safe Care, which is intended to provide infection control and prevention recommendations to outpatient settings. Recommendations include development of an infection prevention program in the facility, specific infection prevention education and training of health care personnel, surveillance of HAIs, and adherence to standard precautions (*19*). This document might serve as a guide to outpatient settings in Los Angeles County and elsewhere for infection prevention practices.

This analysis of outbreak investigations in Los Angeles County demonstrated that HAI outbreaks in outpatient settings occur in diverse settings and can have severe impacts on affected patients and public health. Infection control standards and appropriate event reporting that are promoted, enhanced, and enforced in outpatient settings might help ensure patient safety.
